# Expanding the cardiac phenotype of homozygous *PPA2* variants: insights from a large Finnish family

**DOI:** 10.1186/s13023-026-04523-y

**Published:** 2026-08-13

**Authors:** Krista Heliö, Aurelia Ahokas, Sonja Sulkava, Mikko I. Mäyränpää, Katriina Aalto-Setälä, Sini Weckström, Liliya Euro, Juha Koskenvuo, Anu Suomalainen, Tiina Heliö, Tiina Ojala

**Affiliations:** 1 Heart and Lung Center, ERN GUARD-Heart Center, University of Helsinki and Helsinki University Hospital, Helsinki, Finland; 2 Stem Cell and Metabolism Research Program Unit, Faculty of Medicine, University of Helsinki, Helsinki, Finland; 3 Department of Clinical Genetics, University of Helsinki and HUS Diagnostic Center, University Hospital, Helsinki, Finland; 4 Department of Pathology, HUS Diagnostic Center, University of Helsinki and HUS Diagnostic Center, Helsinki, Finland; 5 Faculty of Medicine and Health Technology, Tampere University, Tampere, Finland; 6 Heart Hospital, Tampere University Hospital, Tampere, Finland; 7 Blueprint Genetics, A Quest Diagnostics Company, Espoo, Finland; 8 HUS Diagnostic Centre, Helsinki University Hospital, Helsinki, Finland; 9 HiLife, Helsinki University, Helsinki, Finland; 10 Pediatric Cardiology, Pediatric Research Center, New Children’s Hospital, University of Helsinki and Helsinki University Hospital, Helsinki, Finland

**Keywords:** PPA2, Cardiomyopathy, Mitochondrial diseases, Sudden cardiac death

## Abstract

**Background:**

Cardiomyopathies in children associate with significant morbidity and mortality. PPA2 deficiency is a rare mitochondrial disorder linked to sudden cardiac death in children and adolescents. Most reported cases are post-mortem, with limited data on surviving individuals. To expand the phenotypic understanding of *PPA2*-related disease, we describe the cardiac features of nine previously unreported homozygous carriers of the *PPA2* c.380G > T, p.(Arg127Leu) variant.

**Methods:**

This study included 23 individuals (22 living, one deceased) from a large Finnish family. All participants underwent genetic testing. Twenty individuals were interviewed by telephone, 16 underwent cardiac evaluation, and hospital records were reviewed for six participants.

**Results:**

Nine individuals homozygous for the *PPA2* p.(Arg127Leu) variant were identified. Two 15-year-old males experienced severe cardiac events following alcohol exposure: one suffered sudden cardiac death, while the other developed fulminant myocarditis. In the deceased male, a recent respiratory infection also preceded the fatal event. The remaining seven homozygotes, aged 24 to 81 years, reported no cardiac symptoms, had limited alcohol exposure restricted to communion, and showed no pathological findings on cardiac evaluation. No participants displayed neurological symptoms attributable to *PPA2*-related disease. All 14 heterozygous carriers were asymptomatic.

**Conclusions:**

PPA2 deficiency represents a rare but potentially fatal cause of sudden cardiac death or fulminant myocarditis, particularly in the setting of alcohol exposure. Our findings indicate that individuals homozygous for the *PPA2* c.380G > T, p.(Arg127Leu) variant may remain asymptomatic into adulthood.

## Introduction

Cardiomyopathies in children are rare but associate with significant morbidity and mortality. Although infrequent, cardiomyopathy may present primarily with sudden cardiac death (SCD) [1, 2]. Typically, one third of the pediatric cardiomyopathy patients receive a genetic diagnosis but there have been some differences between cardiomyopathy subtypes, and diagnostic yield even up to 80% has been reported [3–6].

Mitochondrial disorders are a rare cause of cardiomyopathies, more frequently observed in children, who face a particularly poor overall survival rate, whereas adults may present with a broader or milder spectrum of symptoms [7, 8]. Identifying a mitochondrial disease as the underlying cause of cardiomyopathy is crucial, as it holds significant prognostic and therapeutic implications.

Inorganic phosphatases PPA1 and PPA2 in humans, located in the cytoplasm and mitochondria respectively, are enzymes catalyzing the hydrolysis of pyrophosphate (PPi) into phosphates (Pi). PPi is formed in a variety of nucleotide triphosphate-dependent reactions required for DNA, RNA, protein, and lipid synthesis. PPA2 is necessary for maintenance of the mitochondrial genome and binds to an energy-linked enzyme similar to F0-ATPase [9].

PPA2 deficiency, caused by variants in the mitochondrial pyrophosphatase gene *PPA2* (HGNC:28883), is associated with a rare recessive mitochondrial cardiomyopathy [10, 11]. This deficiency has been linked to sudden cardiac failure syndrome, which is often triggered by viral infections or extreme alcohol hypersensitivity. In majority of the reported cases, patients have succumbed to the disease, with the *PPA2* variant identified post-mortem. Mortality rates are high, and symptoms range from unexpected SCD in infancy to dilated cardiomyopathy (DCM) and life-threatening arrhythmias later in life. Only a small minority of individuals with *PPA2* variants have remained asymptomatic [5, 10–20].

As *PPA2*-associated disease is a newly recognized and rare condition, data on adult patients with homozygous or compound heterozygous *PPA2* variants, particularly those exhibiting a milder spectrum of the disease, remain scarce. However, it is crucial to gather more information about the natural course of the disease in living patients—not only those who have experienced SCD. Such knowledge is vital for improving patient counselling and guidance on treatment decisions.

To expand the phenotypic understanding of *PPA2* variants in adults, we now present the cardiac phenotype of nine previously unreported homozygotes and 14 heterozygotes, all carrying the *PPA2* c.380G > T, p.(Arg127Leu) variant within this single large Finnish family. These findings not only enrich our knowledge of the disease spectrum but also underscore the urgent need for genetic screening in families with unexplained cardiac events.

## Materials and methods

### Participants

This study included 23 participants from one large Finnish family with suspected *PPA2* associated cardiomyopathy. This study had two brothers as index patients, one living and one deceased. The living index patient was recruited from the Pediatric Cardiology Department of the Helsinki University Hospital where all pediatric patients with severe cardiomyopathy in Finland are examined.

### Molecular genetic studies

The living index patient underwent gene panel testing including 58 genes associated with arrhythmias. Subsequently, the identified *PPA2* c.380G > T, p.(Arg127Leu) variant (NM_176869.3, Chr4:106,367,601, GRCh37/hg19) was investigated in 22 participating family members. Sanger sequencing confirmed the presence of the *PPA2* c.380G > T, p.(Arg127Leu) variant in all family members. Genetic testing was conducted by the Blueprint Genetics laboratory (Espoo, Finland). To confirm the homozygous genotype with another method, exome sequencing at the Laboratory of Genetics in the Helsinki University Hospital (Helsinki, Finland) was performed in the mother of the index patients. The pathogenicity of this *PPA2* variant was assessed using the in-house modified classification scheme from the American College of Medical Genetics and Genomics (ACMG) in the Blueprint Genetics laboratory and by the original ACMG guidelines in the Laboratory of Genetics in the Helsinki University Hospital [21].

### Cardiac evaluation

16 participants underwent cardiac evaluation, including 12-lead electrocardiogram (ECG), echocardiography, physical examination, and appropriate laboratory tests. Some participants also underwent cardiac magnetic resonance imaging (CMR), a stress-test, or Holter-monitoring. Additional clinical data were obtained from all available hospital records. Of six participants, only previous hospital records were available. In one deceased patient, data were obtained from the death certificate. Cardiomyopathy was diagnosed following the European Society of Cardiology (ESC) guidelines [22].

To assess possible triggers and neurological symptoms, 20 participants were interviewed via telephone by one of the authors (AA, SS, TH, TO). The same set of questions was used for each participant. The questionnaire included questions about seizures, muscle symptoms, ataxia, balance problems, and atypical symptoms during infections. Available hospital records were also reviewed for any neurological or neuromuscular diagnoses. No systematic formal neurological examination was performed as part of this study. In addition, possible triggers were assessed by including questions about alcohol consumption, infections, fasting, and physical exercise.

Of the deceased patient, cardiac findings and possible triggers were analyzed by interviewing family members and obtaining data from the death certificate. No additional myocardial tissue was available for further pathological assessment.

### Molecular analysis: homology model

To evaluate the structure-functional impact of R127L mutation in human PPA2, a homology model was generated. X-ray crystal structure of human PPA1 enzyme (PDB ID 7btn) was used as a template. Sequences of human PPA1 and PPA2 share 52.8% identity and 67.7% similarity according to BLOSUM62 similarity matrix, justifying reliability of selected template. Protein sequences were aligned using ClustalW algorithm in BioEdit software and pairwise alignment was submitted to Swiss-Model server for homology modelling. PyMol software was used to insert magnesium ions into obtained hPPA2 model using superimposition with the structure of PPA1. To identify PPi-binding site and insert molecule of pyrophosphate into the PPA2 model, a 3D-structure of PPi was retrieved from PubChem database and docking was performed using HDock server.

## Results

**Fig. 1:**
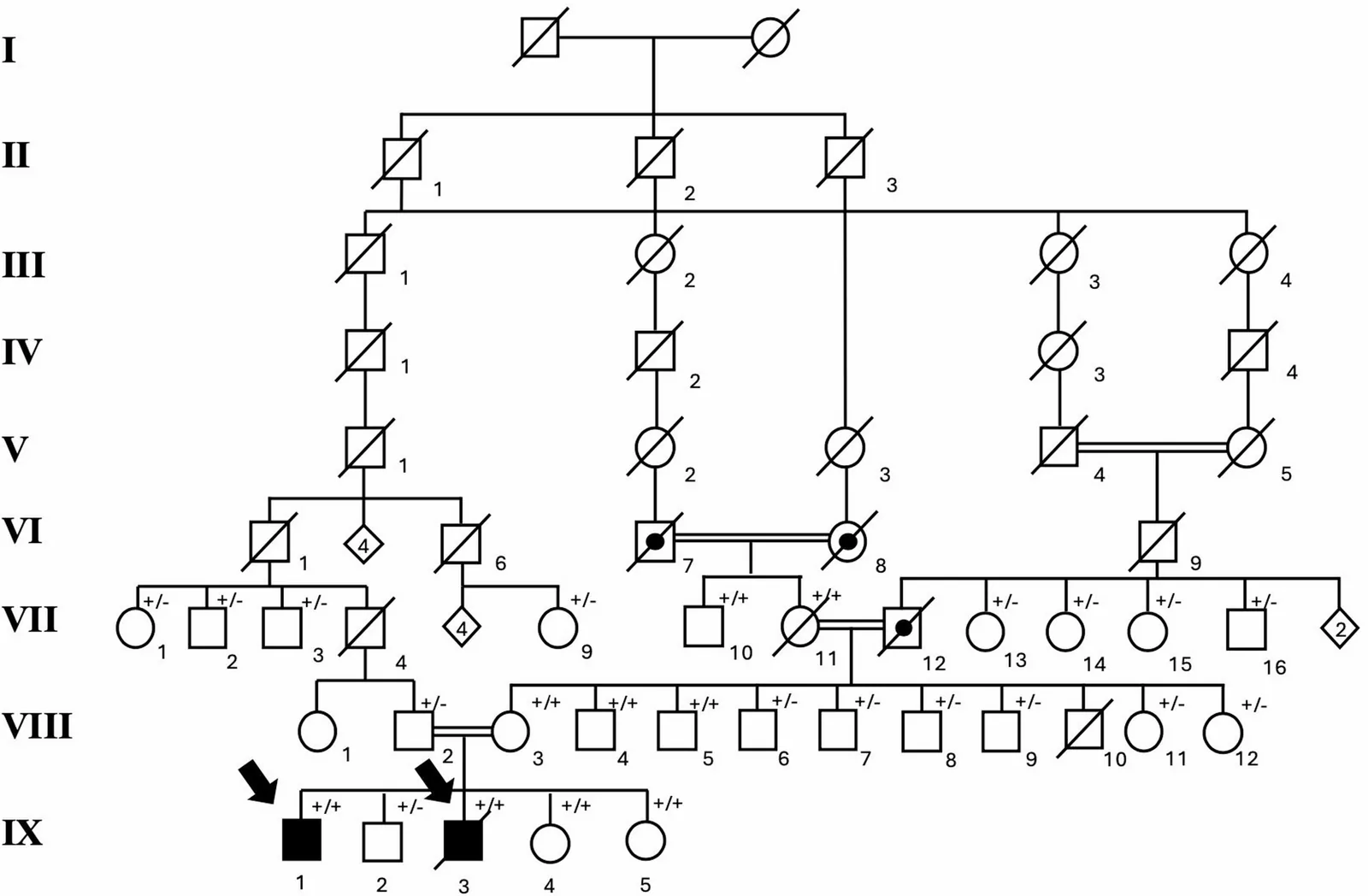
Nine-generation pedigree of the family. Circles represent females, squares males, symbols filled with black indicate cardiomyopathy. Arrow indicates index patients. Consanguineous marriages indicated with double lines between symbols. Genotype marked as +/- for individuals heterozygous for the *PPA2* c.380G > T, p.(Arg127Leu) variant, +/+ for homozygotes, black dot inside the symbol for obligatory carriers

All 23 participants were found to carry the *PPA2* c.380G > T, p.(Arg127Leu) variant, nine were homozygotes and 14 heterozygotes (Fig. 1). The *PPA2* c.380G > T, p.(Arg127Leu) variant is present in heterozygous state in the gnomAD population database at an overall frequency of 0.0004571 and of 0.0001567 in Finnish Europeans. Based on the available evidence, the variant was classified as pathogenic by the Blueprint Genetics Laboratory and as likely pathogenic by Genetic Laboratory of the Helsinki University Hospital.

The median age was 41 years in homozygotes and 59 years in heterozygotes. The only pediatric patients analyzed were the two index patients, the deceased index patient who died suddenly at the age of 15 and the living index patient who was recruited at the age of 14 but reached adulthood during follow-up. Nearly half of the homozygotes (*n* = 4, 44%) and heterozygotes (*n* = 6, 43%) were women. Overall cohort characteristics are outlined in Table 1.

**Table 1:** Cohort demographics and clinical variables

	Homozygote	Heterozygote
**Participants**	9	14
Female	4 (44)	6 (43)
**Age (median)**	41	59
**Age < 18 at diagnosis**	2	0
**Holter (= yes)**	5 (56)	1 (7)
VES > 500	1 (20)	0
SVES > 500	0	0
NSVT	2 (40)	0
FA	1 (20)	0
**SCD**	1 (11)	0
**ICD**	2 (22)	0
**PM**	0	1 (7)
**CMR**	6 (67)	7 (50)
Major LGE	1 (17)	2 (29)
Unspecific LGE	0	4 (57)
**Interview**	7	14
**Other anamnestic information**	1	0
**Alcohol intake**		
Yes	8 (100)	12 (86)
None	0	2 (14)
**Maximal intake**		
0.2 units (communion)	6 (75)	5 (36)
1– 2 units	1 (13)	2 (17)
5–10 units	1 (13)	2 (14)
>10 units	0	3 (21)
**Alcohol intolerance**	2 (25)	1 (7)
**Atypical symptoms during infections**	1 (13)	0

For categorical variables numbers are presented as number (percentage). VES, ventricular extrasystole; SVES, supraventricular extrasystole; NSVT, non-sustained ventricular tachycardia; FA, atrial fibrillation; SCD, sudden cardiac death; ICD, implantable cardiac defibrillator; PM, pacemaker; CMR, cardiac magnetic resonance imaging; LGE, late gadolinium enhancement. One unit of alcohol equals 12 g of pure alcohol

### Index cases

A previously healthy male homozygote (IX:3) suffered a SCD at the age of 15, the cause of death was a mild myocarditis diagnosed at autopsy without genetic examinations. He had consumed approximately six units of alcohol previously. As a child, he had been sensitive to infections and frequently vomited during viral respiratory infections. A week before his death, he had a minor respiratory infection.

Due to the death in the family, the at that time asymptomatic 14-year-old sibling (IX:1) underwent cardiac assessment. In the assessment, he had short-coupled ventricular extrasystoles (VES) and episodes of non-sustained ventricular tachycardia (NSVT). He received an implantable cardioverter-defibrillator (ICD) as primary prophylaxis. Genetic panel testing revealed the homozygous *PPA2* variant, no other pathogenic or likely pathogenic variants were identified in any of the other genes analyzed. After the results from the genetic panel testing, the same homozygous variant was identified post-mortem in the deceased IX:3.

A year later the living index patient experienced chest pain after consuming two units of alcohol. His TnI concentration was markedly elevated (170,859 ng/L, normal < 45 ng/L) and he was hospitalized. CMR showed a slightly enlarged and slightly hypokinetic left ventricle (LVEF 50%) with extensive subepicardial inferolateral late gadolinium enhancement (LGE). In follow-up a month later, ventricular size and function had normalized, but LGE persisted. The clinical diagnosis was fulminant myocarditis. PET-CT was not conducted and no endomyocardial biopsies were taken during the hospitalization. The index patient has since reached adulthood, his condition has remained stable, and to date no shockable rhythms have occurred.

### Homozygous family members

#### Alcohol consumption and symptoms

Seven living homozygous family members were assessed, and six of them were interviewed. All six reported to only ever have consumed alcohol in the context of religious communion, which involves a very small quantity of alcohol (approximately 0.2 units, with one unit defined as 12 g of pure alcohol). None had consumed alcohol otherwise, and none reported any symptoms related to this limited exposure.

#### Cardiac symptoms and hospitalizations

Two homozygous family members had presented to a hospital due to chest pain. A 21-year-old sibling of the index patient (IX:5) experienced chest pain during a COVID-19 infection; however, both ECG and troponin levels were within normal limits. Another homozygote (VII:11), aged 80, experienced an episode of chest pain associated with mildly elevated troponin I levels (250 ng/L; normal < 45 ng/L), ST-segment depressions on ECG, and reported muscle weakness in the upper limbs. The underlying cause remained unexplained, as coronary angiography did not reveal significant coronary artery disease.

#### Cardiac imaging

Five homozygous family members underwent echocardiography, which showed normal left ventricular size and function in all individuals. One participant, who had a diagnosis of hypertension, was found to have a mildly thickened interventricular septum measuring 15 mm. CMR was performed in five homozygous individuals, all of whom demonstrated normal ventricular size, function, and no pathological LGE.

#### Electrocardiographic findings and arrhythmias

Resting ECGs were normal in all homozygous family members. Holter-monitoring was performed in four individuals. Three had normal results, while one exhibited frequent VES (more than 500 in 24 h), NSVT, and episodes of atrial fibrillation. One homozygous family member had received an ICD, though no data were available regarding arrhythmic events preceding implantation.

#### Neurological and muscular findings

None of the homozygous family members reported experiencing atypical symptoms during infections. Three reported occasional muscle spasms. One individual had a history of myoclonic epilepsy diagnosed in childhood. No other neurological or neuromuscular disorders were reported.

### Heterozygous family members

#### Alcohol consumption and symptoms

Fourteen heterozygous family members were interviewed, of whom 12 (86%) reported alcohol consumption. Five had consumed only communion wine, involving a minimal amount of alcohol. Two individuals had consumed 1–2 units of alcohol, two had consumed between 5 and 10 units, and three had consumed more than 10 units on a single occasion. One heterozygous individual reported experiencing palpitations after alcohol intake, although no arrhythmias were documented. No other symptoms associated with alcohol consumption were reported.

#### Cardiac symptoms and hospitalizations

One heterozygote had been hospitalized with myocarditis following a respiratory infection. None of the other heterozygous family members had been hospitalized for infection-related complications.

#### Cardiac imaging

Five heterozygous individuals underwent echocardiographic evaluation, all showing normal left ventricular size and systolic function. CMR revealed normal ventricular size and function in all heterozygous individuals examined. Two individuals exhibited striated intramyocardial or midmyocardial LGE in the interventricular septum. Additionally, three had a mildly thickened septum (10–13 mm) with nonspecific LGE in the basal septum.

#### Electrocardiographic findings and arrhythmias

Standard ECGs were normal in all heterozygous individuals. None of the heterozygous individuals had a history of ventricular arrhythmias. Holter monitoring was performed in one individual and showed normal findings. One individual had received a pacemaker due to sinus node dysfunction.

#### Neurological and muscular findings

None of the heterozygous individuals reported neurological conditions, muscle cramps, myoclonus, ataxia, or balance disturbances. No epilepsy or neuromuscular disorders were reported in this group.

#### Molecular analysis

**Fig. 2:**
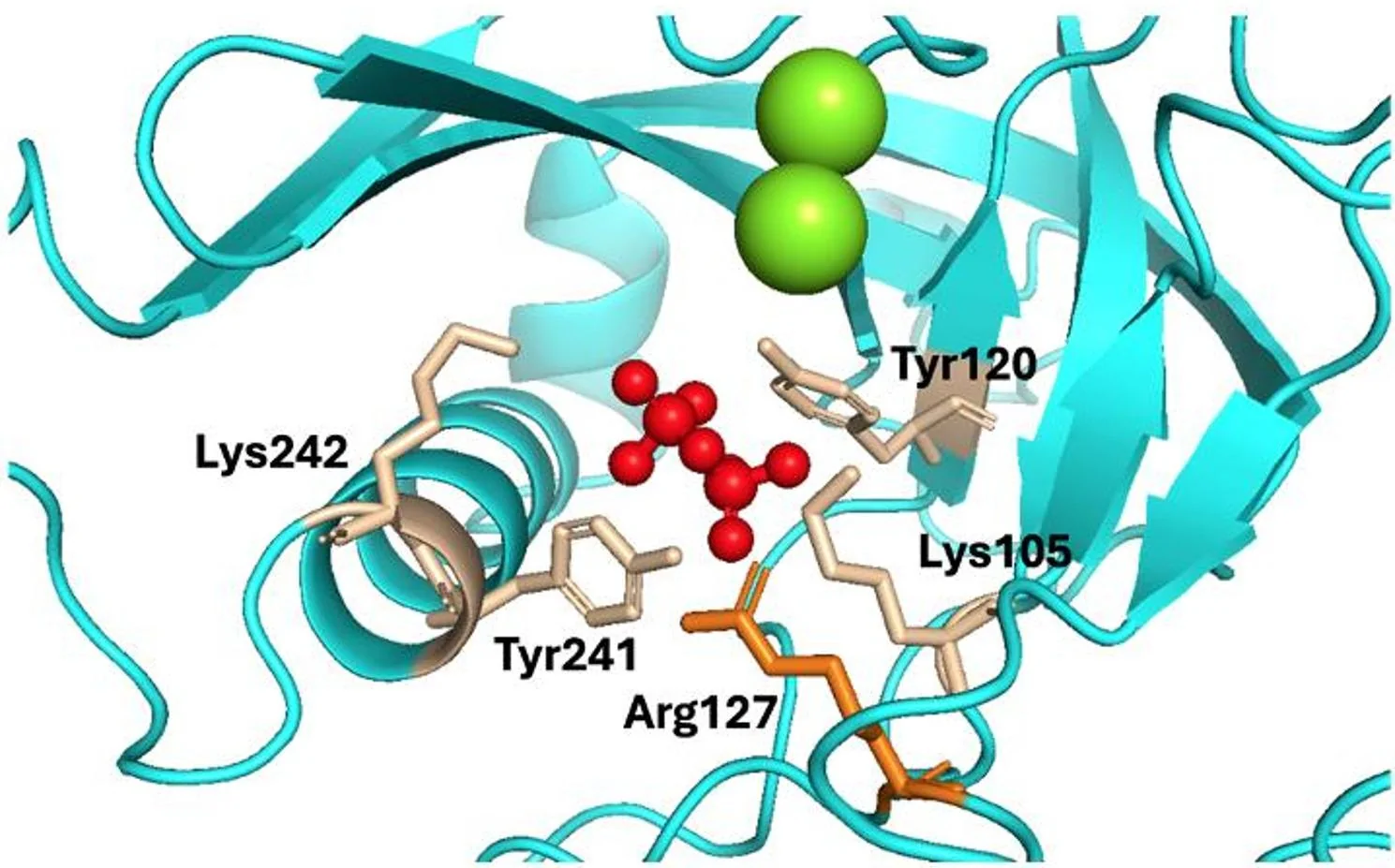
Modelled hPPA2 with R127 (orange) located within active site. Docked pyrophosphate is shown in red, two magnesium ions are in green

**Fig. 3:**
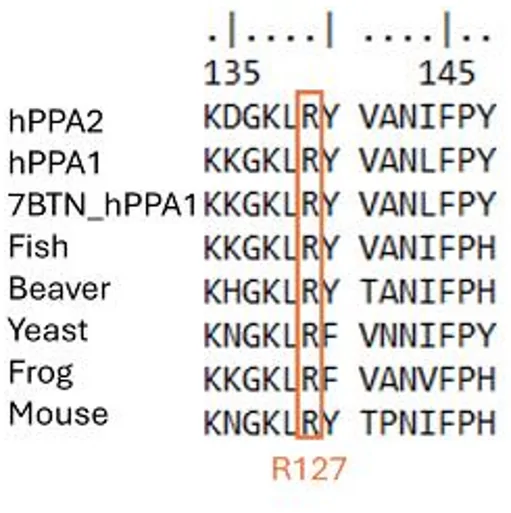
R127 is strictly conserved across the evolution

The PPA2 homology model revealed R127 to locate within the active site of the enzyme within 7 Å from PPi and near the invariant catalytic amino acid residues. (Fig. 2) Multiple sequence alignment of PPA2 homologs revealed that arginine 127 is fully conserved across evolution indicating its indispensable role in protein function (Fig. 3). Analysis of pathogenicity degree of R127L variant using PolyPhen-2 and SIFT databases predicted it to be damaging. REVEL score was 0.641 [23–25]. As most of previously studied variants in the active site of PPA2 resulted in decreased catalytic activity, R127L most likely has similar effect.

## Discussion

In this study, we report a range of phenotypes associated with the homozygous and heterozygous *PPA2* c.380G > T, p.(Arg127Leu) variant. Unlike previous reports in which most individuals were deceased and often presented with SCD in infancy or adolescence, our cohort primarily consisted of living individuals, including adults, allowing for a broader perspective on the phenotypic variability and potential modifying factors associated with this variant.

As summarized in Tables 2 and 3, including our cohort, altogether 78 individuals homozygous or compound heterozygous for *PPA2* variants have been reported. Most reported deaths occurred in children under the age of two or in adolescents aged 14 years and older. Notably, no reported deaths have occurred in individuals aged 3–13 years. In younger children, viral infections were the predominant trigger, whereas in adolescents and adults, alcohol consumption has consistently been reported as a precipitating factor [5, 10–20]. In our cohort, this pattern was corroborated: two homozygous adolescents experienced severe cardiac events following alcohol intake—one of them suffered SCD and the other developed fulminant myocarditis and ventricular arrhythmias. Alcohol exposure appears to be an important trigger for severe cardiac events in adolescents and adults with biallelic *PPA2* variants. However, in the deceased index patient, a recent respiratory infection preceded the fatal event, and therefore alcohol exposure may not fully explain the clinical manifestation. The possibility that alcohol and infection or other metabolic stressors act together to precipitate myocardial injury or arrhythmia should be considered. In summary, *PPA2*-associated disease carries a significant risk of SCD, with distinct age-related peaks in occurrence.

**Table 2:** Summary of all reported individuals homozygous or compound heterozygous for PPA2 variants, including this cohort

Age	≤ 2 years	3–13 years	14–17 years	≥ 18 years	Total
**Total patients**	41	5	14	18	*78*
**Deceased (%)**	41 (100)	0	12 (86)	2 (11)	*55 (71)*
female/male	18/23	0	7/5	1/1	*26/29*
**Alive (%)**	0	5 (100)	2 (14)	16 (89)	*23 (29)*
female/male	0	3/2	2/0	10/6	*15/8*
**Trigger for symptoms**	viral infection	viral infection	alcohol consumption	alcohol consumption, viral infection, both	
**Cause of death**	SCA, cardiogenic shock	-	SCA, cardiogenic shock	SCA	
**References**	[5, 10, 11, 14, 16, 19]	[13, 16]	[10, 12, 16–18]	[10, 16, 18, 20, 26–28]	

SCA, sudden cardiac arrest

**Table 3:** Summary of clinical features in all reported living individuals homozygous or compound heterozygous for PPA2 variants, including this cohort

	≤ 2 years	3–13 years	14–17 years	≥ 18 years
**Number of participants**	0	5	2	16
Female	0	3	2	10
Male	0	2	0	6
**Clinical features**				
DCM/dilated ventricle	0	4	0	4
Recorded NSVT, VT, or VF	0	2	1	4
Myocardial fibrosis and/or inflammation (histology or CMR)	0	3	2	8
Myocarditis	0	0	1	4
Neurological symptoms	0	2	0	3
No findings or symptoms	0	1	0	5
**Trigger for symptoms**				
Alcohol consumption	0	0	2	5
Recent viral infection	0	2	0	1
Both	0	0	0	3
**References**		[13, 16]	[12, 17]	[10, 16, 18, 26–28]

DCM, dilated cardiomyopathy; NSVT, non-sustained ventricular tachycardia; VT, ventricular tachycardia; VF, ventricular fibrillation; CMR, cardiac magnetic resonance imaging

In our study, except for the two index patients, nearly all other homozygous participants had either completely avoided alcohol throughout life or had only consumed minimal amounts (less than one unit), and none reported symptoms related to this limited alcohol exposure. Heterozygous individuals, including those who reported consuming alcohol in moderate to high quantities, did not experience symptoms or arrhythmic events. This supports the idea that *PPA2*-related disease is inherited in a recessive manner: homozygosity for the p.(Arg127Leu) variant may confer a higher sensitivity to alcohol-triggered cardiac events, and the phenomenon is not observed in heterozygotes.

The mechanism of SCD in PPA2 deficiency is likely multifactorial. Ventricular ectopy, NSVT, ICD implantation, and reported ventricular tachyarrhythmias suggest that malignant ventricular arrhythmias may be an important final pathway. At the same time, myocarditis-like presentations, markedly elevated troponin concentrations, LGE on CMR, and autopsy findings of myocardial inflammation or fibrosis reported in previous studies indicate that arrhythmias may occur in the setting of acute myocardial injury or a pre-existing myocardial substrate [5, 10–14, 16–18, 20]. Mitochondrial energetic failure may further lower the threshold for electrical instability during metabolic stress [29]. Thus, current data do not support a purely arrhythmogenic or purely inflammatory mechanism; rather, sudden death may result from the interaction between impaired mitochondrial reserve, acute triggers, myocardial injury, and ventricular arrhythmia susceptibility. Further research is still needed on the subject.

In contrast to earlier studies where viral illness often precipitated fatal cardiac decompensation in infants, our findings indicate that such vulnerability does not extend universally to older homozygotes [5, 10–14, 16, 18, 19]. Recently, adults homozygous or compound heterozygous for *PPA2* variants have also been reported to develop cardiac symptoms following viral illness [26–28]. One of them was a 58-year-old woman homozygous for the p.(Arg127Leu) variant who suffered heart failure after a febrile infection. CMR showed extensive fibrosis and her systolic function later normalized in follow-up. (27) In our study, apart from the one deceased homozygote, who died following a viral infection and alcohol consumption, none of the other homozygous individuals demonstrated increased sensitivity or atypical symptoms related to infections. Even the patient who experienced chest pain during a COVID-19 infection had no biochemical or imaging evidence of myocardial injury. Guimier et al. previously conducted a functional analysis on *E.Coli* that showed lower mitochondrial pyrophosphatase activity in recombinant PPA2 variants when compared to wild-type PPA2. (16) Interestingly, the variant found in our family had the lowest pyrophosphatase activity of all tested disease-causing variants in *PPA2*. In their study it was hypothesized that the individuals with pathogenic variants in *PPA2* could be sensitive to hyperthermia as it could lead to further reduction in the already reduced enzyme function [16]. As the residual PPA2 activity might differ between *PPA2* variants, the sensitivity to hyperthermia or infections could also differ. The role of febrile infections alone as a trigger for cardiac events, especially in older individuals, remains to be explored.

PPA2 deficiency is considered a mitochondrial disorder because *PPA2* encodes the mitochondrial inorganic pyrophosphatase that hydrolyses pyrophosphate generated during biosynthetic reactions. Impaired PPA2 activity is expected to disturb mitochondrial homeostasis and reduce cellular energetic reserve [9]. The predominantly cardiac phenotype associated with PPA2 deficiency may reflect the exceptionally high and continuous energy demand of cardiomyocytes, their dependence on oxidative phosphorylation, and the limited capacity of the myocardium to tolerate abrupt mitochondrial dysfunction [29]. Under basal conditions, residual PPA2 activity and compensatory mechanisms may be sufficient in some individuals. However, additional stressors such as alcohol exposure, infection, fever, inflammation, fasting, or other forms of metabolic stress may exceed this reserve and lead to acute myocardial injury, arrhythmias, or cardiac failure. This stress-dependent model could partly explain the incomplete penetrance observed in our cohort.

In the deceased index patient, the available autopsy data indicated mild myocarditis. No additional myocardial tissue was available for ultrastructural examination, respiratory-chain enzyme studies, immunohistochemistry, or other mitochondrial investigations. Therefore, the present study cannot directly demonstrate mitochondrial dysfunction in cardiac tissue. Nevertheless, previously reported *PPA2* cases have described myocardial inflammation, fibrosis, cardiomyopathy, and acute cardiac failure, findings that are compatible with vulnerability of the myocardium to impaired mitochondrial function and metabolic stress [10–20, 26, 27].

Cardiac imaging in our cohort showed that five of eight living homozygous individuals had completely normal findings, and none fulfilled diagnostic criteria for cardiomyopathy. Notably, LGE in CMR—a surrogate for myocardial fibrosis—was present in only one homozygous individual, the same patient who had suffered myocarditis. This contrasts with previous reports, in which myocardial fibrosis or inflammation was commonly identified, even in asymptomatic individuals. For instance, three previously reported living patients aged 17 to 38 years were found to have LGE on CMR despite being clinically asymptomatic [10, 12]. Similarly, autopsy reports in deceased patients have described varying degrees of myocardial fibrosis or inflammation [5, 10–14, 16–18, 20]. In the light of these findings, even though some patients may show signs of fibrosis, a normal CMR does not exclude the possibility of *PPA2* variant homozygosity.

Heterozygous individuals in our cohort were largely asymptomatic, with normal ECGs, Holter recordings, and cardiac imaging. Although some showed mild nonspecific CMR findings such as septal thickening or striated intramyocardial LGE, none had functional cardiac impairment or arrhythmic events. These findings align with previous reports indicating that heterozygous carriers of *PPA2* variants typically do not experience SCD or overt cardiomyopathy.

This *PPA2* c.380G > T, p.(Arg127Leu) variant has previously been reported in 11 individuals, in three in homozygous and in eight in compound heterozygous state. Of the compound heterozygous carriers, one individual remained asymptomatic at the age of 10, the other seven died between four months and two years of age [10, 11, 14, 16]. In one previously reported family two siblings homozygous for the p.(Arg127Leu) variant suffered a SCD after consuming alcohol [16]. To our knowledge, we report the largest group of living individuals homozygous for *PPA2* variant, with the same gene variant, described to date. Prior to this study, only 15 of 69 reported individuals with biallelic *PPA2* variants had survived into adolescence or adulthood. Most of these survivors had exhibited some degree of neurological or cardiac pathology. For example, Guimier et al. described six individuals aged 4 to 40 years with multisystemic manifestations including mitochondrial dysfunction, arrhythmias, and progressive cardiomyopathy, while Zhao et al. reported a 5-year-old girl with seizures and heart failure linked to a viral illness [13, 16]. To our knowledge, only one previously reported patient—a 10-year-old girl with a compound heterozygous genotype—was noted to be completely asymptomatic, making the largely benign presentation in our adult homozygous participants particularly notable [16].

One of the most striking findings of this study is the apparent stability of the cardiac phenotype over time in those who have reached adulthood. Despite concerns about progressive myocardial involvement, our data suggest that significant deterioration is not inevitable, and in fact, most homozygotes in our cohort remained stable, especially in the absence of known triggers such as alcohol consumption. This supports the hypothesis that lifestyle and environmental exposures may play a critical role in phenotypic expression.

The relatively benign course observed in several adult homozygous family members suggests that homozygosity for the *PPA2* c.380G > T, p.(Arg127Leu) variant alone may not be sufficient to determine clinical outcome. Strict avoidance of alcohol is likely to be an important protective factor, but it may not fully explain the striking difference between this family and previously reported cohorts. Because the asymptomatic homozygotes belong to the same extended family, shared genetic background or protective genetic modifiers segregating within the family could contribute to the observed incomplete penetrance. Potential modifiers could involve mitochondrial biogenesis and respiratory-chain reserve, cardiomyocyte energy handling, alcohol metabolism, inflammatory responses to infection, or arrhythmia susceptibility. Epigenetic and environmental factors may also influence clinical expression. However, this study did not include genome-wide modifier analysis or functional testing; therefore, this interpretation remains speculative and requires further investigation.

Although this study is limited by its small sample size and retrospective data collection in some cases, it highlights the importance of early identification and lifestyle counselling—especially regarding alcohol avoidance—in individuals with biallelic *PPA2* variants. To clarify the mechanisms by which environmental triggers interact with the underlying genetic mitochondrial susceptibility to produce severe phenotypes, further research, including longitudinal follow-up and functional studies, is essential.

### Limitations

The cohort was small, complete data were not available on all participants. Clinical data presented in this study has partly been collected from previous health records. No systematic formal neurological examination was performed as part of this study; therefore, subtle neurological manifestations cannot be fully excluded. Even though all available hospital records were gathered, as some patients lived in other hospital districts, we did not have access to the complete data.

## Conclusions

Although *PPA2* associated mitochondrial cardiomyopathy is rare, it should be considered a potential cause of SCD or fulminant myocarditis. Alcohol appears to be an important trigger in adolescents and adults with biallelic *PPA2* variants, however, the relative contribution of concomitant factors, such as infection, inflammation, or fever, remains uncertain. Our findings support the notion that individuals heterozygous for the *PPA2* p.(Arg127Leu) variant are not at risk of clinical disease, but the disease follows a recessive inheritance pattern. Some individuals homozygous for the *PPA2* c.380G > T, p.(Arg127Leu) variant may remain mostly asymptomatic and live a normal life. Regular clinical follow-up of homozygous individuals is recommended, as the natural history of the disease remains poorly understood.

## Data Availability

The datasets analyzed during the current study are not publicly available due to the need to maintain the privacy of the individuals who participated in the study and to comply with the GDPR legislation but are available from the corresponding author on reasonable request.

## Abbreviations

ACMG: American College of Medical Genetics and Genomics
CMR: Cardiac magnetic resonance imaging
DCM: Dilated cardiomyopathy
ECG: Electrocardiogram
ESC: European Society of Cardiology
ICD: Implantable cardioverter-defibrillator
LGE: Late gadolinium enhancement
NSVT: Non-sustained ventricular tachycardia
Pi: Phosphate
PPi: Pyrophosphate
SCD: Sudden cardiac death
VES: Ventricular extrasystole
